# Thrust enhancement and degradation mechanisms due to self-induced vibrations in bio-inspired flying robots

**DOI:** 10.1038/s41598-023-45360-4

**Published:** 2023-10-25

**Authors:** Dipan Deb, Kevin Huang, Aakash Verma, Moatasem Fouda, Haithem E. Taha

**Affiliations:** University of California Irvine, Irvine, CA 92697 USA

**Keywords:** Biotechnology, Engineering

## Abstract

Bio-inspired flying robots (BIFRs) which fly by flapping their wings experience continuously oscillating aerodynamic forces. These oscillations in the driving force cause vibrations in the motion of the body around the mean trajectory. In other words, a hovering BIFR does not remain fixed in space; instead, it undergoes oscillatory motion in almost all directions around the stationary point. These oscillations affect the aerodynamic performance of the flier. Assessing the effect of these oscillations, particularly on thrust generation in two-winged and four-winged BIFRs, is the main objective of this work. To achieve such a goal, two experimental setups were considered to measure the average thrust for the two BIFRs. The average thrust is measured over the flapping cycle of the BIFRs. In the first experimental setup, the BIFR is installed at the end of a pendulum rod, in place of the pendulum mass. While flapping, the model creates a thrust force that raises the model along the circular trajectory of the pendulum mass to a certain angular position, which is an equilibrium point and is also stable. Measuring the weight of the BIFR and the equilibrium angle it obtains, it is straightforward to estimate the average thrust, by moment balance about the pendulum hinge. This pendulum setup allows the BIFR model to freely oscillate back and forth along the circular trajectory about the equilibrium position. As such, the estimated average thrust includes the effects of these self-induced vibrations. In contrast, we use another setup with a load cell to measure thrust where the model is completely fixed. The thrust measurement revealed that the load cell or the fixed test leads to a higher thrust than the pendulum or the oscillatory test for the two-winged model, showing the opposite behavior for the four-winged model. That is, self-induced vibrations have different effects on the two BIFR models. We felt that this observation is worth further investigation. It is important to mention that aerodynamic mechanisms for thrust generation in the two and four-winged models are different. A two-winged BIFR generates thrust through traditional flapping mechanisms whereas a four-winged model enjoys a clapping effect, which results from wing-wing interaction. In the present work, we use a motion capture system, aerodynamic modeling, and flow visualization to study the underlying physics of the observed different behaviors of the two flapping models. The study revealed that the interaction of the vortices with the flapping wing robots may play a role in the observed aerodynamic behavior of the two BIFRs.

## Introduction

Bio-inspired flying robots (BIFRs), more specifically Flapping Wing Micro Air Vehicles (FWMAV), have been a major focal point of research in the aerodynamics, dynamics, and control community in the last few decades. In the twentieth century, the main attention was directed toward uncovering the unconventional lift mechanisms in flapping flight. With the more precise observation of the insect flight and how they make use of the unsteady lifting mechanisms (e.g., wake capture, leading-edge vortex, etc.), this puzzle was resolved^1,2,3^. Having understood the lifting mechanisms in insect flight, several researchers independently developed unique designs for FWMAVs^4,5^. Zakaria et al. (2015) showed that the inclusion of inertial power requirements is essential for physical and proper optimization^6^. They further studied the aerodynamic forces generated in forward flight for different Reynolds numbers and flapping frequencies^7^. Whitney^8^ designed and developed one of the tiniest FWMAVs. Keennon et al.^9^ developed the AeroVironment Nano Hummingbird as a hovering ornithopter. TU Delft researchers designed, developed, and studied the aerodynamic and dynamic performances of the Delfly^10,11^. Any design, like the previously mentioned ones, is aimed at a particular objective or flying condition like forward flight, hovering, etc.

One of the most challenging flying conditions of flapping flight is hovering. There have been numerous studies to investigate the aerodynamics of hovering flapping flight. Weis-Fogh tested the quasi-steady assumption for insect flight, where unsteady effects are more conspicuous, and showed that quasi-steady aerodynamics can predict the main features of hovering flights^12^. However, Ellington^13^ examined the results of Weis-Fogh’s in the light of more accurate kinematic and morphological data, and his conclusion was opposite to that of Weis-Fogh’s. Ellington^14^ further showed that the leading edge separation bubble plays a prominent role in the hovering flight of insects. In comparison to a thin airfoil, Ellington asserted that the leading edge bubble modifies the camber and the thickness of the thin profile which enhances lift at low Reynolds numbers. Bayiz et al.^15^ compared the hovering efficiency in rotary and flapping modes using rigid rectangular wings. They observed that flapping wings are more efficient in achieving a higher average lift coefficient in hovering. Sarkar et al.^16^ studied aerodynamic performance under asymmetric flapping kinematics using sinusoidal and triangular waveforms. The frequency-asymmetry mechanism showed an increase in aerodynamic loads for the sinusoidal case. During the faster stroke, the lift can be enhanced depending on the level of asymmetry. The results of these investigations can be used to design an efficient flapping robot for hovering.

Discussion on hovering insects is incomplete without pondering upon the question of stability. Sun et al.^17^ found that the pitching moment produced by a change in horizontal speed is the primary source of an unstable oscillatory mode, whereas vertical force produced by changes in vertical speed is the primary source of a stable slow subsidence mode. These results are mainly based on averaging the flight dynamics over the flapping cycle. In contrast, using chronological calculus a hidden stabilization mechanism was discovered in a hovering hawkmoth. Taha et al.^18^ showed that insects use a passive stabilization mechanism through their natural wing oscillation; this is called vibrational stabilization. It is a natural phenomenon observed in systems like the Kapitza pendulum^19^. A bio-inspired flapping robot in two degrees of freedom system can exhibit vibrational stabilization as well^20^.

It is important to emphasize that when an insect hovers over a flower, it is not completely stationary in space over the flower. Instead, it undergoes oscillatory motion in almost all directions. These oscillations can be observed in the video, Hedrick and Daniel (2006) presented in the supplementary section of their paper^21^. Hence, it experiences self-induced vibrations. This vibration may change the flow field around the wing and thus affect the generated aerodynamic forces. In ideal hovering, there should be no self-induced vibration but in real cases, these vibrations are unavoidable due to the inevitable oscillatory nature of the driving aerodynamic forces. To the authors’ best knowledge, there is little effort exerted that focuses on this point^22,23^, and the effect of self-induced vibration on the clapping mechanism is significantly under-explored. The current work is dedicated to studying the effect of self-induced body vibration in the flapping flight. To achieve this goal, we considered two different setups for aerodynamic force measurement (specially thrust) : (1) Pendulum setup or oscillatory test and (2) Load cell setup or fixed test. In the pendulum setup, we replaced the mass of a pendulum with a flapping wing robot. Whenever the robot flaps, it generates thrust and moves upward along the circular trajectory of the pendulum, assuming equilibrium at some angular position. We can measure this angle and use it to calculate the average thrust. We prefer this pendulum setup over the Harvard Robofly experimental setup (moving along vertical rails)^24^ because it allows multiple equilibrium positions at different flapping frequencies. Hence, the thrust can be estimated via a simple measurement of the angular position of the pendulum rod. In contrast, if the thrust of a flapping robot moving along vertical rails is different from its weight, the robot will continuously move up (or down); and estimating the thrust will require accurate measurement of the acceleration/deceleration of the robot. Clearly, measuring the angular position of the pendulum rod is simpler and more accurate than measuring the acceleration of a vibrating body. Also, this is a standard setup used to measure thrust generated by rotary wings^25^. The flapping robot vibrates about the angular position i.e., the measured thrust includes the effect of vibration. In contrast, in the loadcell or fixed test setup, there is no room for such a vibration. So using these two setups, we can measure the effect of self-induced vibration on flapping thrust generation.

**Figure 1:**
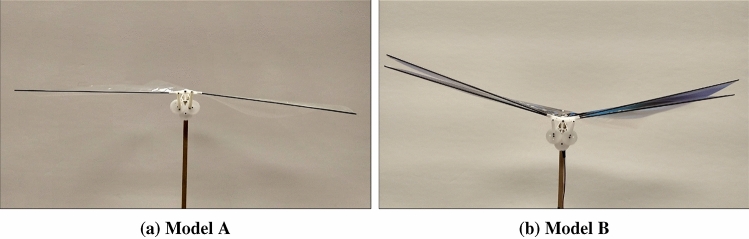
View from the front of the robots which were also used by Balta et al.^35^.

**Figure 2:**
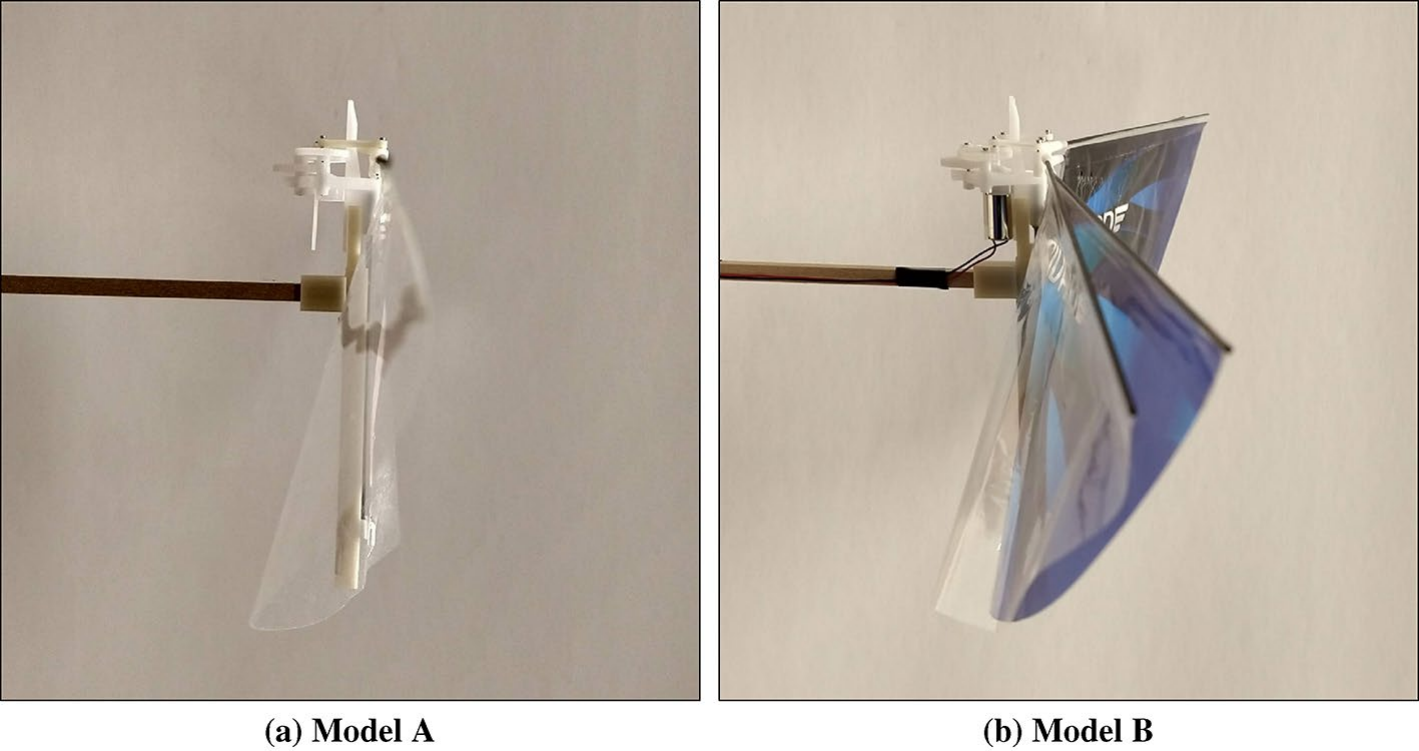
View from the side of the FWMAVs^35^.

**Figure 3:**
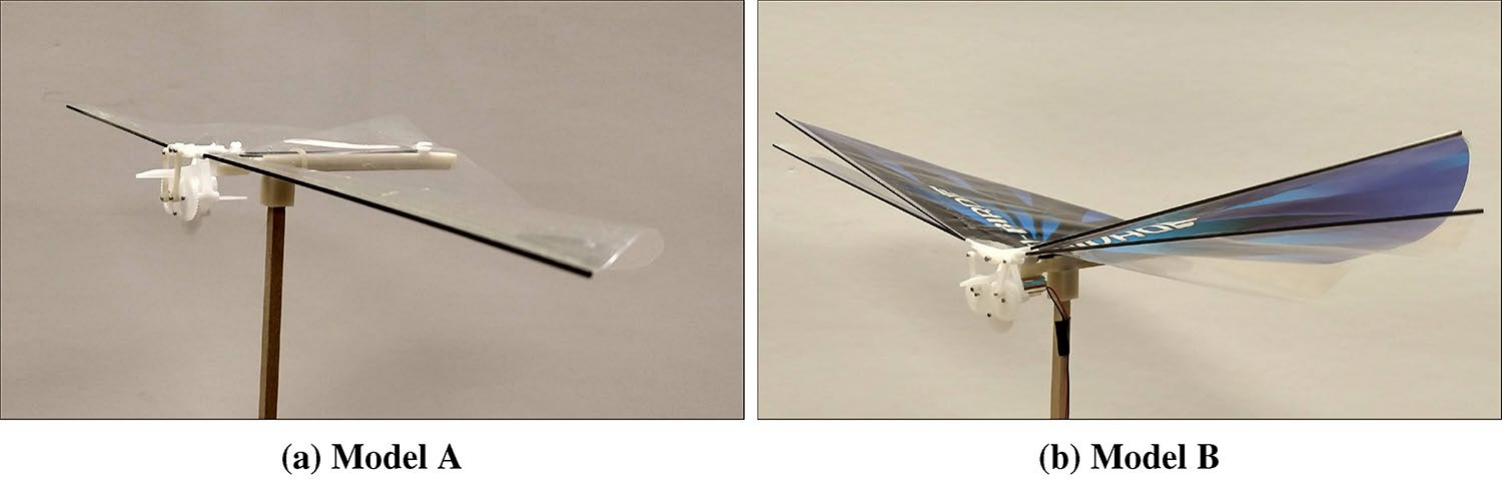
View from the corner of the FWMAVs^35^.

**Figure 4:**
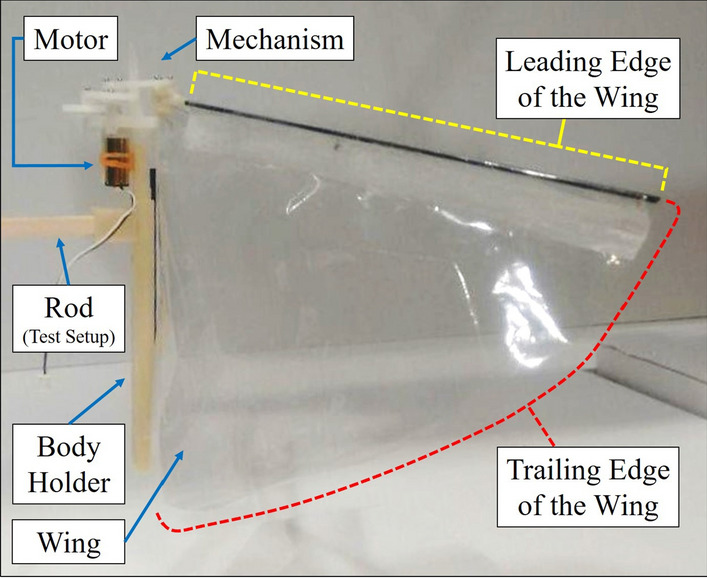
Detailing and different components of Model A^35^.

Two flapping wing robots are considered for this study: one has two wings and the other has four wings. The one, that is two-winged, is named Model A (Figs. 1a, 2a, 3a, 4). The wings of these models have a stroke angle of $\sim 60^{0}$. Unsteady responses like leading edge vortex^1^, wake capture^2,26^ are utilized by the two-winged model in flapping flight. The four-winged model or Model B (Figs. 1b, 2b, 3b) exploits a wing-wing interaction phenomenon named ’clap-and-peel’ for generating thrust. There has been a surge of interest in recent years to study the interaction of multiple bodies in a fluid flow^27–29^. Deb et al.^28^ observed in-phase and out-of-phase oscillations in a couple of rigid plates which are oriented one beside the other, through wind tunnel experiments. They explained the modes of oscillation of the plates through flow field data and visualization. Some studies also included flexibility. Flexible flags in different orientations like tandem and side-by-side and their interaction in those configurations were studied by Alben^30^. In flapping flight, wing-wing interaction has been exploited by the four-wings model by using 'clap-and-fling'^31^ (for flexible wings -'clap-and-peel'^32^) to generate thrust. Outcomes of wing-wing interaction like stronger leading edge vortex^33^ and jet effect^31,34^ are utilized for thrust generation by the clap-and-peel mechanism. Balta et al.^35^ showed with flow visualization that the peeling phase of the flapping cycle draws air in and the clap phase propels it downstream; that thrust is augmented using a jet effect. A blob of air flows between the wings in the peeling phase, which strengthens the leading edge vortex. Some efforts were made to capture the clapping effect into an aerodynamic model^36–38^. Armanini et al.^33^ studied the improved strength of the leading edge vortex during clapping and included this phenomenon in a quasi-steady aerodynamic model. They further studied the clapping-effect interaction with the tail-wake and wing-wake^32^.

The mechanism for thrust generation in a four-winged robot is different from the conventional one due to the wing-wing interaction, or the clap-and-peel mechanism^35,34^. Thus it is expected that the effects of the self-induced vibration will not be similar for the two-winged and the four-winged robots.

In the present work, we investigated the effect of self-induced body vibration on two-winged and the four-winged (clap-and-peel) flapping robots using two different thrust measurement setups, flow visualization, motion capture, and theoretical aerodynamic modeling.

## Observation and experimental setup

The focus of this research is to investigate the effect of self-induced oscillations on two distinct mechanisms of thrust generation for FWMAVs. The flapping robots, Model A (Figs. 1a, 2a, 3a) and Model B (Figs. 1b, 2b, 3b), utilize a crank-rocker mechanism for flapping, which is also used in the same laboratory by Balta et al. (2021)^35^. In the case of Model B, wing-wing interaction takes place which exploits the clap-and-peel mechanism. The motion of the wings moving towards each other during the flapping cycle is called ’clap’, and the ensuing motion of moving away from each other is known as ’peel’. Given the flexibility of the wings, they peel away in this motion and hence the name. Figure 4 shows the leading edge (LE), trailing edge (TE), motor, and other parts of the mechanism.

**Figure 5:**
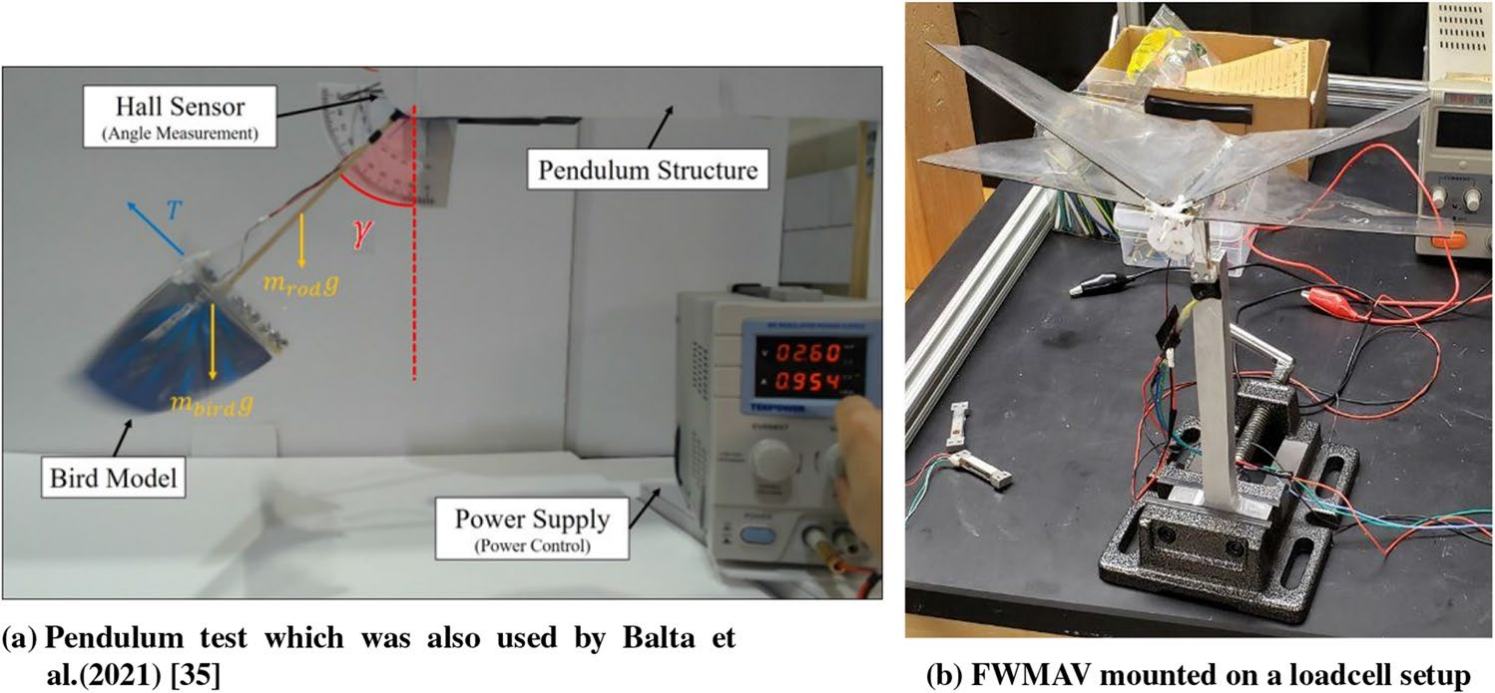
The different setups for aerodynamic force measurements.

The present work is dedicated to testing and analyzing Models A and B. We use different experimental setups like (1) Pendulum test or oscillatory test (Fig. 5a) and (2) Loadcell or fixed test (Fig. 5b) for measuring the thrust generated by the two above-mentioned models. We use uxcell 100g loadcell, with 1mV/V sensitivity. Each run includes a one-second record of data with a sampling frequency of 5000 samples/second. The data is averaged over the maximum number of integer cycles within the data acquisition time span of one second. FFT is then performed for the time series of the measured thrust to estimate the flapping frequency. We trim an aluminum block to fit the loadcell onto it and we mount the FWMAV on the loadcell. The thrust signals from the loadcell are filtered twice before analysis. A hardware low pass filter of type USBPGF-S1 is used for the first stage of filtration. Subsequently, the signals are acquired by the NI DAQ and filtered again by a digital filter provided by the LabVIEW software itself. The same LabVIEW program helps to record the data as well. We have also included the structural response of the loadcell system in the appendix^39^.

The model on the pendulum setup (Fig. 5a) is connected to a rigid wooden rod. When the power supply is turned off or no power is applied to the model, it can relax and assumes a resting state. When the FWMAV attains a certain flapping frequency, it generates thrust and moves upward along the circular arc of the pendulum, as shown in Figure 5a. So, it attains a stable equilibrium point at some angle $\gamma$. The pendulum angle $\gamma$ can be measured using an encoder. It is a digital encoder of type CUI AMT10 with a resolution of 2048 (i.e., 0.18 degrees). By measuring the mass of the wooden pendulum rod (denoted by $m_{r}$) and of the FWMAV (denoted by *m*) we can calculate the average thrust over the flapping cycle. We obtained equation (1) for average thrust calculation with moment balance about the pendulum hinge. The measurement of the damping coefficient of the pendulum hinge is discussed in the appendix^39^.

1 $$\begin{aligned} T = \left( m+\frac{1}{2}m_\mathrm{{r}} \right) g \sin \gamma , \end{aligned}$$

Using this method and the equation we can measure the mean thrust for a given value of the flapping frequency. A stroboscope is used to measure this frequency. In this test, the FWMAV body is free to oscillate. So, the effect of body-induced vibrations is already included in the measured average thrust.

On the other hand, the load cell test (Fig. 5b) measures thrust with time during the flapping cycle. By applying the time average to the obtained data, we can calculate the mean thrust at each given value of the flapping frequency. The loadcell setup consists of a uni-axial loadcell.

**Figure 6:**
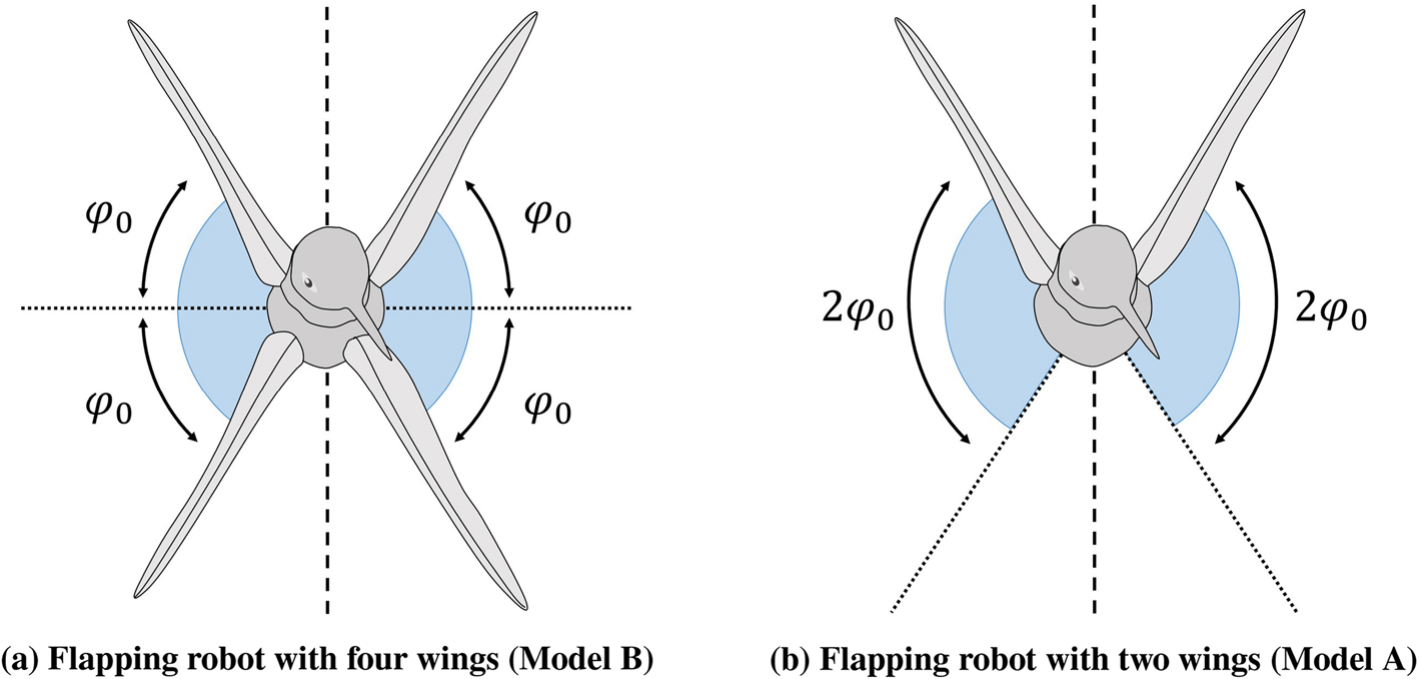
Amplitude of flapping angle of an individual wing corresponding to the flapping robot^35^.

We non-dimensionalize the measured thrust for dynamic similarity. However, it is important to note that the generated thrust depends on the angle swept by the wings, wing surface area, flapping frequency, number of wings, and wingspan. So, we non-dimensionalize the thrust force by $\frac{1}{2}\rho V_{ref}^2 S N$, where $V_{ref}=2\pi f R \Phi$ is a reference speed, taken here as the maximum speed of the wing tip, similar to helicopters and propellers. Also, *f* denotes flapping frequency, *R* denotes wing span and $\Phi$ is the amplitude of the flapping angle for a single wing. Figure 6a shows that angle swept by one wing for Model B is almost half as much for its Model A equivalence shown in Fig. 6b. So, $\Phi =2\phi _{0}$ and $N=2$ are for Model A and similarly we can say that $\Phi =\phi _{0}$ and $N=4$ are for Model B. For Model A the coefficient of thrust is defined as

2 $$\begin{aligned} C_{T}=\frac{T}{\frac{1}{2}\rho (2\pi f R 2\phi _{0})^2 2 S } \end{aligned}$$

whereas, for Model B it can be written as,

3 $$\begin{aligned} C_{T}=\frac{T}{\frac{1}{2}\rho (2\pi f R \phi _{0})^2 4 S } \end{aligned}$$

**Figure 7:**
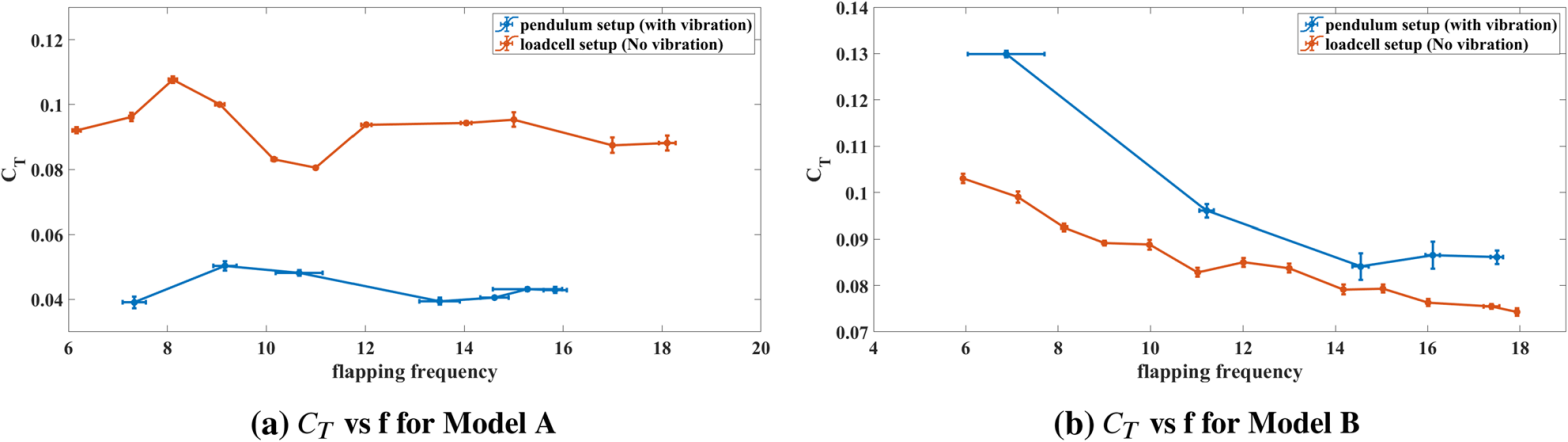
Comparison of thrust co-efficient for both the Models (A and B) from both the pendulum and loadcell setups with error bars.

Figure 7 shows the variations of thrust coefficient with the flapping frequency of three different runs for the two (Fig. 7a) and four-winged (Fig. 7b) flapping models using both the loadcell (fixed test) and pendulum (oscillatory test) setups. Each run is performed at the same input power to the flapping mechanism. The error bars in both the frequency (horizontal bar) and thrust measurements (vertical bars) are presented. It is noted that the horizontal error bars in the pendulum setup are relatively larger than the loadcell setup, which is perhaps due to the less accurate measurement of the flapping frequency using the strobe light. However, it is within reasonable bounds and was deemed satisfactory for the current study. As for the observation, Fig. 7a shows the averaged thrust coefficient $C_{T}$ measured for model A for given flapping frequencies. It clearly shows that the thrust measurements from the oscillatory test are less than the fixed test. On the other hand, the situation is reversed for Model B as shown in Fig. 7b. This clear difference in behavior is the main focus of this paper.

The generated aerodynamic forces by the FWMAVs are periodic in nature. Hence even after achieving a stable equilibrium in the system, the FWMAV oscillates about that equilibrium point. On the other end, the fixed test setup allows no such oscillation. Moreover, Model B exploits wing-wing interaction or clapping effect to generate thrust which differs from the thrust generation of Model A^35^. Thus the vibration also has different effects on the thrust generation and results in the opposite trend observed in Fig. 7.

**Figure 8:**
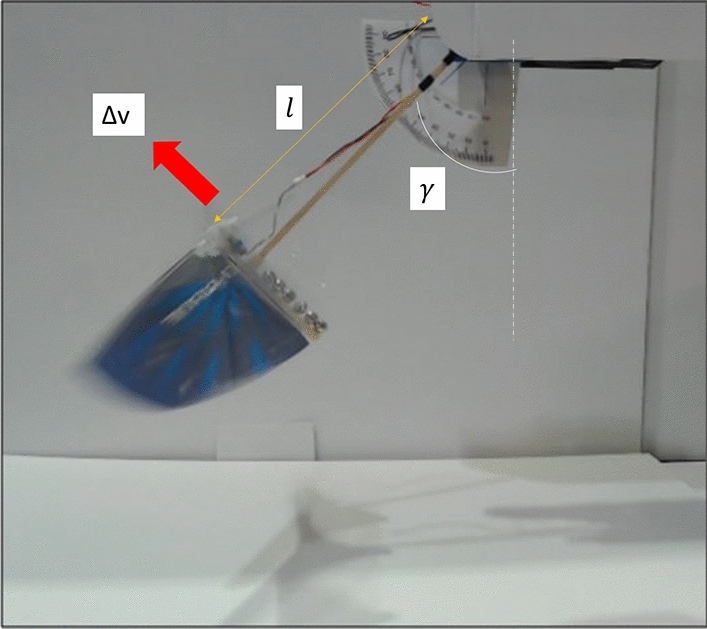
$\Delta v$ measurement from the Pendulum angle.

**Figure 9:**
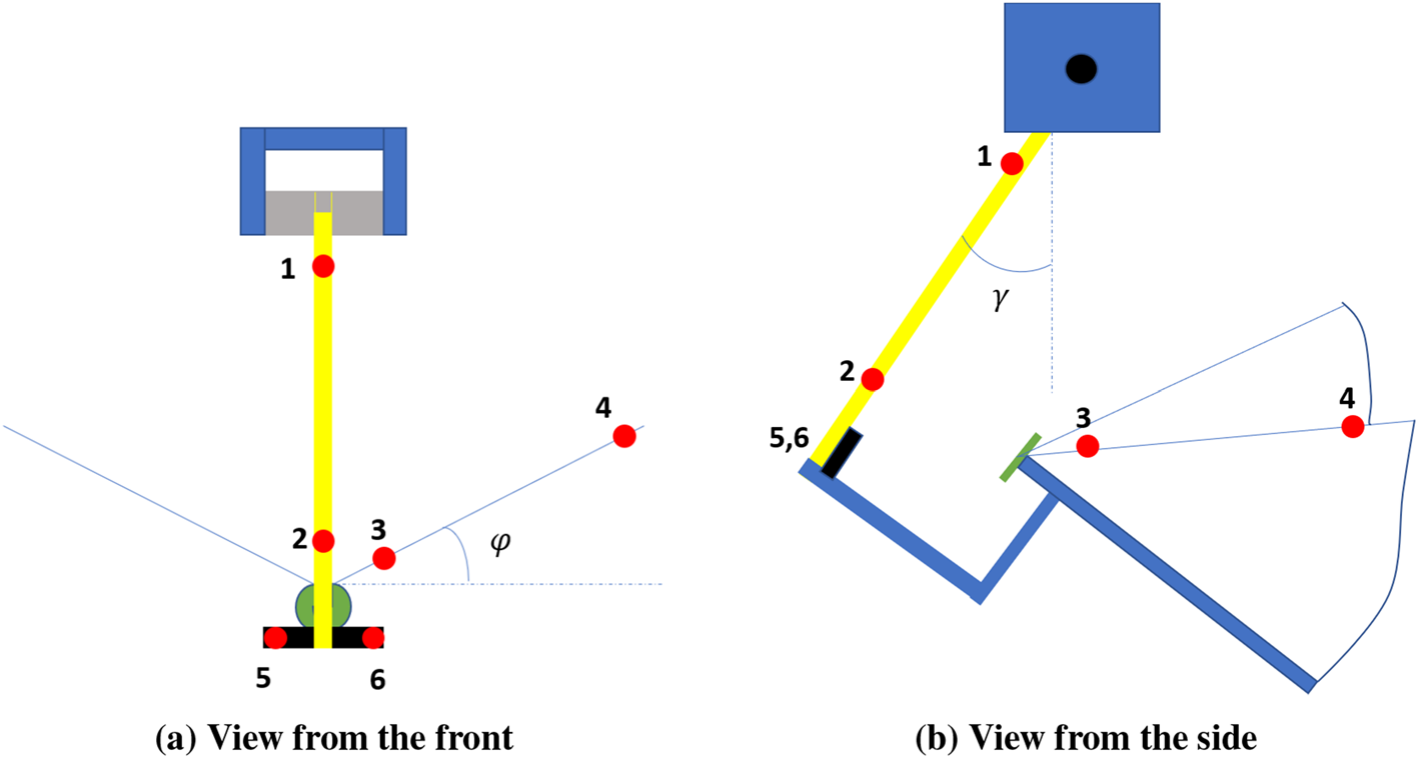
Schematic of the FWMAV and the pendulum with the active markers.

In order to investigate the effect of the vibration, we need to define it first. In the oscillatory test, the angular position of the FWMAV, denoted by $\gamma$, oscillates around a mean point $\gamma _{0}$, with a zero-mean periodic variation ${\widetilde{\gamma }}$. For a given flapping frequency we can say that the FWMAV assumes an angular location $\gamma (t) = \gamma _{0}+{\widetilde{\gamma }}(t)$. Denoting the length of the wooden rod as *l*, the vibration velocity can be defined as $\Delta v= l {\dot{\gamma }}$. The red arrow shows the direction of positive $\Delta v$ in Fig. 8. The flapping angle $\phi$ is also measured simultaneously with the pendulum angle $\gamma$ at a given point in time. For this purpose, we use a motion capture system with one tracker and six markers as mentioned in this 2022 conference paper^40^. Figure 9 shows the positions of these markers. Figure 9a shows that the markers positioned on the wooden rod (1 and 2) are for measuring $\gamma (t)$, the markers on the leading edge (3 and 4) as shown in Fig. 9a are for the measurement of $\phi (t)$, and the two markers (5 and 6) at the bottom of the rod are for defining local horizon. All these markers are active in nature. A 3D tracker receives signals from the markers and sends them to the computer. The tracker has 0.1mm spatial and $1\mu s$ temporal accuracy. The VZSoft software acquires the signal and records the data on the computer. We use MATLAB to read and process the data to observe the perturbation motion.

**Figure 10:**
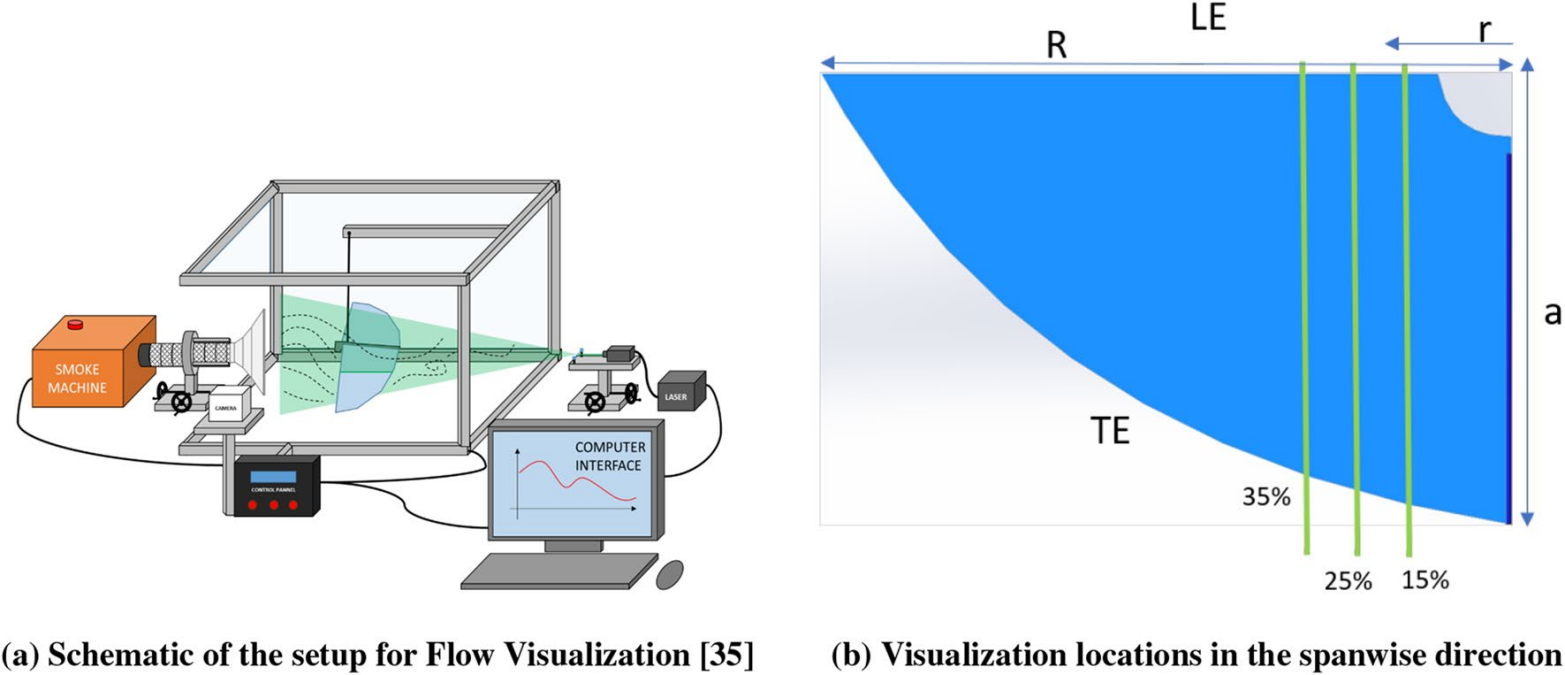
Schematic of the Flow Visualization setup and the spanwise sections on the wing for visualization.

Flow visualization is executed to investigate the effect of the vibration in the flow field for both the models and the setups. The images captured for the cases with and without vibration, explain the flow physics underlying the performance observed in Fig. 7. Figure 10 shows a schematic of the flow visualization setup. Figure 10a shows the FWMAV being mounted near a diffuser, that is attached to a smoke machine. The diffuser is used to inject fog by the machine into the flow. The fog follows the flow field generated by the flapping of the robots. Specific planar sections of the flow field were enlightened by a laser sheet generated by a class III laser machine. The visualization is captured for 6*Hz* flapping frequency with a camera at 240 FPS. As the laser sheet is two-dimensional, the visualization is done at different spanwise positions. These locations are demarcated in green in Fig. 10b.

## Aerodynamic modeling

To further understand the effect of perturbation velocity on flapping wing models (Model A and Model B), it may be prudent to develop an aerodynamic model for both models, which is the focus of this section. The backbone of the adopted aerodynamic model was proposed by Berman and Wang^41^, who were studying the energy-minimizing kinematics in hovering insect flight. However, this aerodynamic model only applies to FWMAV with two wings (Model A), i.e., no wing-wing interactions. Armanini et al.^33^ extended the applicability of this model to FWMAVs with four wings (Model B), by including the clapping effect.

### Aerodynamic model for 2 wings

**Figure 11:**
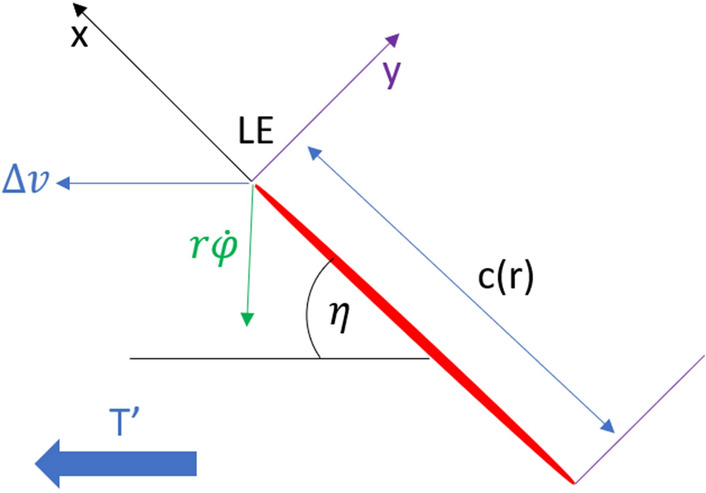
Cross section of the flapping wing at distance r from the body.

Figure 11 shows a cross section (blade element) of a wing at distance *r* from the body of the FWMAV. The red portion represents the chord of the section *c*(*r*) and (*x*, *y*) makes the reference frame at the leading edge (denoted as LE) of the blade element. The green and blue arrows at the leading edge denote the direction of perturbation velocity (i.e., due to vibration) $\Delta v$ and the flapping velocity $r{\dot{\phi }}$, respectively. The thrust generated per unit span is denoted as $T^{\prime }$ and can be expressed by the force generated per unit span in the *x* and *y* directions, $F^{\prime }_{x}$, $F^{\prime }_{y}$ and the pitching angle $\eta$

4 $$\begin{aligned} T^{\prime }=dT/dr= F^{\prime }_{x} {\text {cos}} \eta -F^{\prime }_{y} {\text {sin}} \eta \end{aligned}$$

According to Berman and Wang^41^ the forces $F^{\prime }_{x}$ and $F^{\prime }_{y}$ can be expressed in terms of the bound vortex $\Gamma$, added masses $m_{11}$ and $m_{22}$, the velocity and acceleration components $v_{x}$, $v_{y}$, $a_{x}$ and $a_{y}$ and the viscous forces in those directions $F^{\prime v}_{x}$ and $F^{\prime v}_{y}$

5 $$\begin{aligned} F^{\prime }_{x}=-\rho \Gamma v_{y}-m_{11} a_{x}-F^{\prime v}_{x} \end{aligned}$$

6 $$\begin{aligned} F^{\prime }_{y}=\rho \Gamma v_{x}-m_{22} a_{y}-F^{\prime v}_{y} \end{aligned}$$

The bound vortex $\Gamma$ can be written in terms of the translational and rotational coefficients $C_{t}$ and $C_{R}$, the total velocity $|v|=\sqrt{v^{2}_{x}+v^{2}_{y}}$, as well as the angle of attack $\alpha$ and pitching rate ${\dot{\eta }}$. The relation is shown in equation (7).

7 $$\begin{aligned} \Gamma =-\frac{1}{2} C_{t} c(r)|v| {\text {sin}} 2 \alpha +\frac{1}{2} C_{R} c^{2}(r) {\dot{\eta }} \end{aligned}$$

The viscous force $F^{\prime v}$ are given by

8 $$\begin{aligned} F^{\prime v}=\frac{1}{2} \rho c(r) C_{D}|v|<v_{x}, v_{y}>\end{aligned}$$

The added mass terms are given by

9 $$ \begin{aligned} m_{11}=\frac{1}{4} \pi \rho a^{2} \; \; \; \; \; \;  \&  \; \; \; \; \; \; m_{22}=\frac{1}{4} \pi \rho c^{2}(r) \end{aligned}$$

The coefficient of drag is written as

10 $$\begin{aligned} C_{D}=2 C_{t} \sin ^{2} \alpha \end{aligned}$$

The angle of attack $\alpha$ is given by

11 $$\begin{aligned} \alpha =\tan ^{-1}\left( \frac{v_{y}}{v_{x}}\right) \end{aligned}$$

Where the velocity and the acceleration components are given as

12 $$\begin{aligned} v_{x}= & {} -r {\dot{\varphi }} {\text {Sin}} \eta \end{aligned}$$

13 $$\begin{aligned} v_{y}= & {} -r {\dot{\varphi }} {\text {Cos}} \eta \end{aligned}$$

14 $$\begin{aligned} a_{x}= & {} \dot{v}_{x} \end{aligned}$$

15 $$\begin{aligned} a_{y}= & {} \dot{v}_{y} \end{aligned}$$

$\phi (t)$ is the flapping angle of the wing which is measured using the previously mentioned motion capture system. The pitching angle of the wing $\eta (r,t)$ is a function of *r* (spanwise location) and *t* (time). The pitching angle is modeled as a Fourier series in time since the motion is periodic, with spatially varying coefficients to account for wing flexibility.

16 $$\begin{aligned} \eta (r, t)={} & {} a_{0}(r)+a_{1}(r) \cos (2 \pi f t)+b_{1}(r) \sin (2 \pi f t)+a_{2}(r) \cos (4 \pi f t) +b_{2}(r) \sin (4 \pi f t) \end{aligned}$$

Cubic polynomials are assumed for these coefficients as

17 $$\begin{aligned} a_{i}= & {} A_{i 1} r+A_{i 2} r^{2}+A_{i 3} r^{3} \end{aligned}$$

18 $$\begin{aligned} b_{i}= & {} B_{i 1} r+B_{i 2} r^{2}+B_{i 3} r^{3} \end{aligned}$$

**Figure 12:**
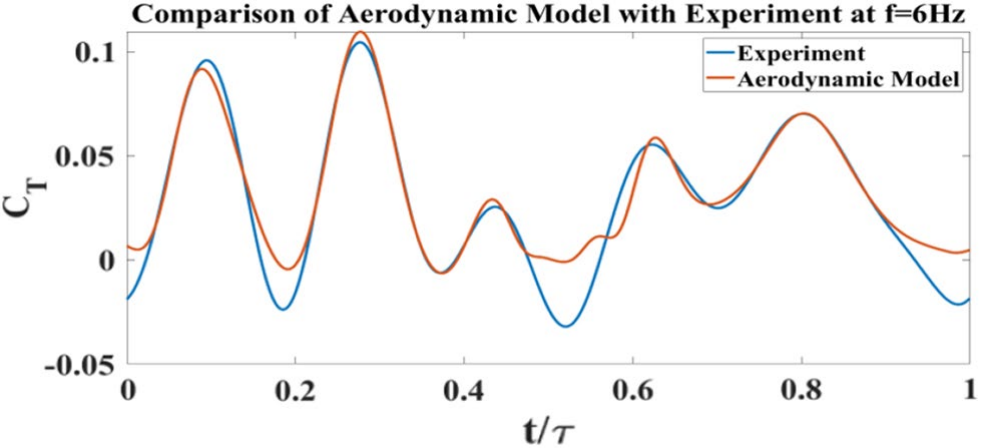
Comparison of aerodynamic model results with experiment at 6 Hz for 2wings.

After obtaining thrust per unit span $T'$ from equation (4), it is then integrated over the span of the wing to determine total thrust generated by the FWMAV and then normalized to obtain the coefficient of thrust according to equation (2).

Upon defining the complete structure of the aerodynamic model, some unknown parameters must be specified. These include $A_{01}, A_{02}, A_{03}, A_{11}, A_{12}, A_{13}, A_{21}, A_{22}, A_{23}, B_{11}, B_{12}, B_{13}, B_{21}, B_{22}, B_{23}, C_{t}  \&  C_{R}$. They are determined by formulating an optimization problem to minimize the error between the theoretical prediction and the experimental measurements of thrust time-variation over the cycle using the same kinematics. The results from this optimization problem are shown in Fig. 12, which compares the optimized aerodynamic model with experimental measurements (the load cell test data, shown in Fig. 5b, are used in this case). As can be seen, the resulting coefficient of thrust from the model and from the experimental setup has a close match over the majority of the cycle. The measured perturbation velocity $\Delta v$ can be applied to the model to study the effect of induced vibrations during the flapping cycle. To apply this perturbation, we need to modify the components $v_{x}$ and $v_{y}$ of each airfoil section to account for the contribution of $\Delta v$. The modified velocities are written as,

19 $$\begin{aligned} v_{x}=\Delta v {\text {Cos}} \eta -r {\dot{\varphi }} {\text {Sin}} \eta \end{aligned}$$

20 $$\begin{aligned} v_{y}=-\Delta v {\text {Sin}} \eta -r {\dot{\varphi }} {\text {Cos}} \eta \end{aligned}$$

### Aerodynamic model of 4 wings

In the case of the four-winged robot, the aerodynamic model is almost the same as that of the two-winged robot with a few extensions to capture the effect of wing-wing interaction. During the ’peel’ motion, a suction is created between the wings, which sucks air from the ambient towards it and strengthens the leading edge vortex. Therefore, the generated circulation is modified in a way that empirically captures the change in the strength of the leading edge vortex.

21 $$\begin{aligned} \begin{aligned} \Gamma&=-\frac{1}{2} C_{t} c(r)|v| {\text {Sin}} 2 \alpha +\frac{1}{2} C_{F} c^{2}(r) {\dot{\eta }}_{\text{ fling } }, \quad {\dot{\eta }}_{\text{ fling } }>0 \\ \Gamma&=-\frac{1}{2} C_{t} c(r)|v| {\text {Sin}} 2 \alpha +\frac{1}{2} C_{R} c^{2}(r) {\dot{\eta }} \end{aligned} \end{aligned}$$

Also, the added mass term $m_{22}$ is modified to take into account the ’peeled away’ portion of the chord^33^.

22 $$\begin{aligned} m_{22}=\frac{1}{4} \pi \rho c_{e f f}^{2}(r,t) \end{aligned}$$

**Figure 13:**
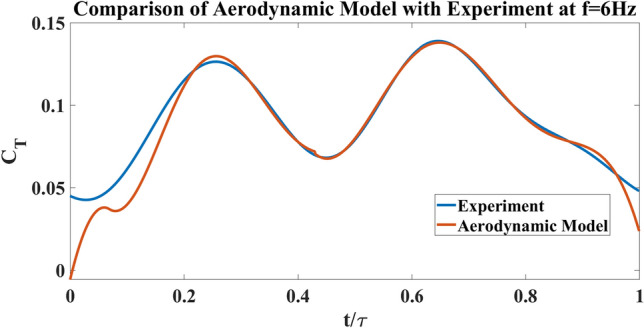
Comparison of aerodynamic model results with experiment at 6 Hz for 4wings.

The result of the match of the optimization formulation is shown in Fig. 13. Similar to the previous case, the measured $\Delta v$ can be applied to this model to analyze the effect of vibration during the flapping cycle.

## Results and discussion

In this section, we focus on the behavior of the flapping models at one flapping frequency (6*Hz*) to present a more complete picture than just inspecting thrust measurements. We scrutinize the flow field using flow visualization along with measuring the oscillatory motion of the body and feeding it into the presented aerodynamic model to gain some insight into the observed behaviors presented in Fig. 7. While the selected frequency should be representative of the considered regime, there is no guarantee that the explanations suggested in this section for the 6*Hz* case will hold exactly at other frequencies. However, we expect the pictures to be reasonably similar. The spanwise sections of 25% and 35% of the wing are chosen for flow visualization of Model A. The sectional flapping velocities at the above mentioned locations are presented simultaneously with the perturbation velocity at a given point during the flapping cycle. For Model B, 15% spanwise position is chosen and similar flow field images and velocities are investigated. Reference speed $V_{ref}=2\pi f R \Phi$ is used to non-dimensionalize both the sectional velocity $r\dot{\phi }$ and the induced velocity $\Delta v$.

### Effects of self-induced vibrations on the two-wings model (Model A)

**Figure 14:**
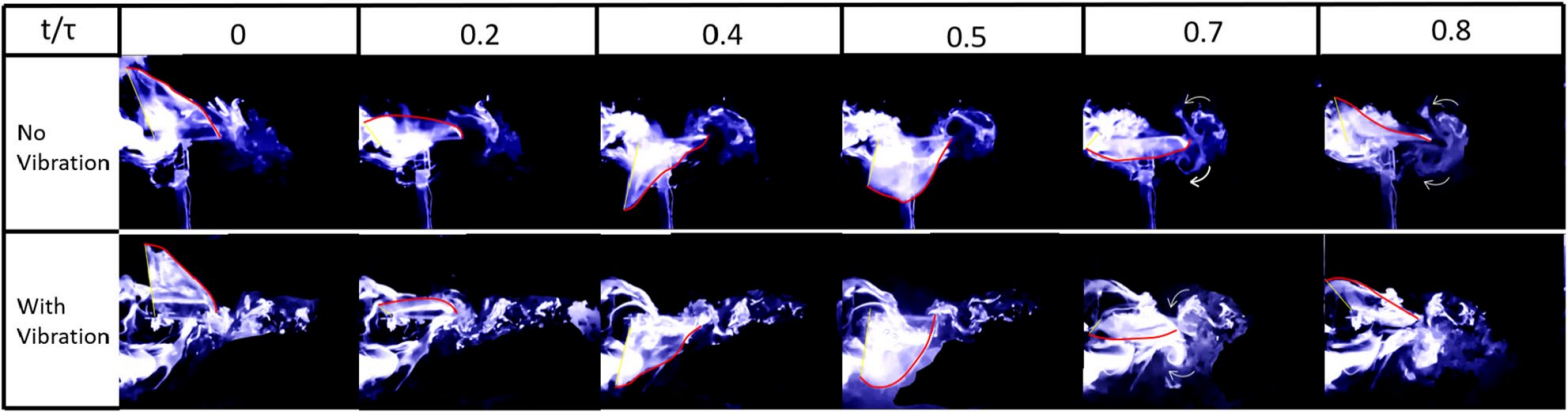
Flow visualization images from oscillatory test (with vibration) and fixed test (no vibration) at 25% spanwise location for Model A.

**Figure 15:**
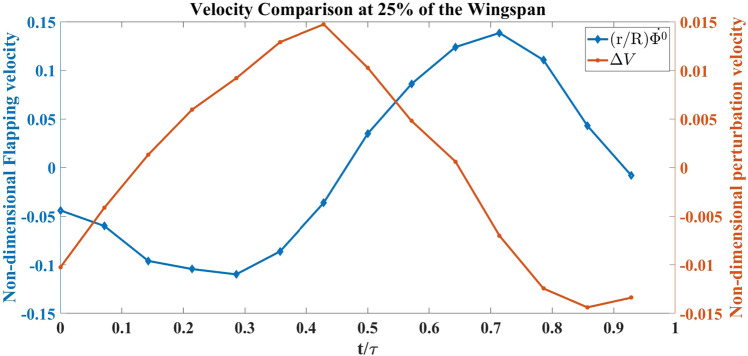
Normalized vibration and flapping velocity at 25% spanwise location for Model A.

**Figure 16:**
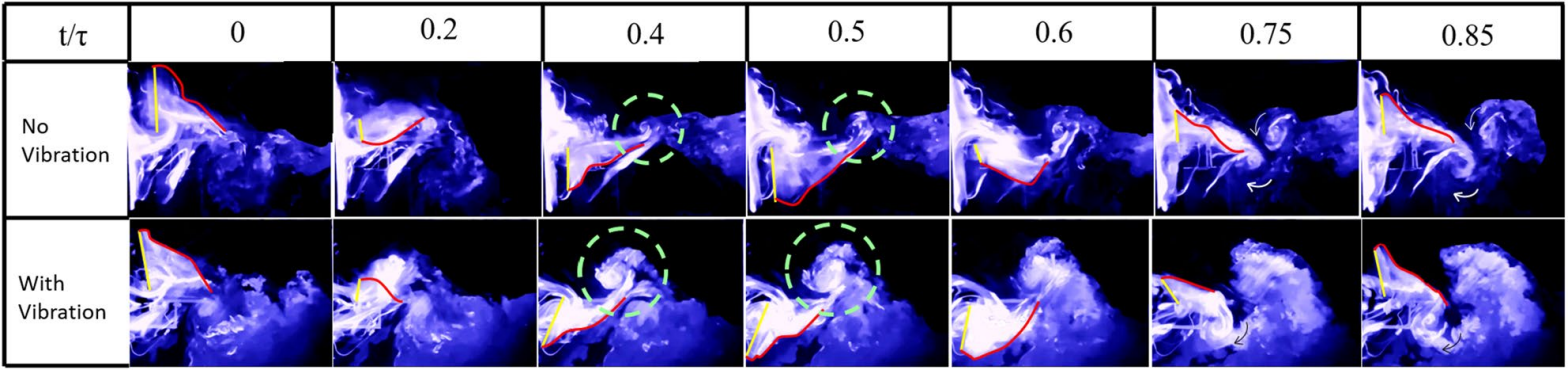
Flow visualization images from oscillatory test (with vibration) and fixed test (no vibration) at 35% spanwise location for Model A.

The images presented in Fig. 14 primarily compare the flow fields produced by the flapping of Model A, at 25% spanwise location: $1^{st}$ row is without any perturbation and the $2^{nd}$ row is with the self-induced vibrations, shown in Fig. 15. Both cases are compared at 6*Hz* flapping frequency (*f*). The row presents flow fields at a given time during the flapping period. The time parameter is denoted by *t*, whereas $\tau$ is the time period of flapping. In the figures (Figs. 14, 16 and 19) red shows the trailing edge outline and yellow is used for the leading edge. Fig. 15 shows the comparison between non-dimensional flapping velocity and normalized perturbation velocity at 25% wingspan. The flapping cycle begins at $t/\tau =0$, when the wing starts its down-stroke near the maximum angle of flapping. The wing finishes down-stroke around $t/\tau =0.5$ and ensues into upstroke.

**Figure 17:**
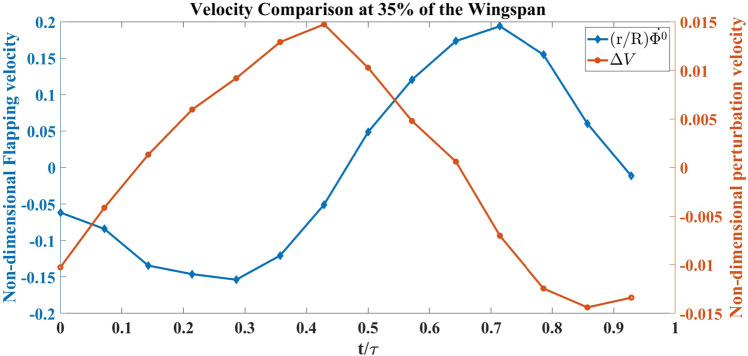
Normalized vibration and flapping velocity at 35% spanwise location for Model A.

Figure 14 shows a couple of vortices with opposite rotations near the TE for all the cases at the instant $t/\tau =0.7$. The white arrows denote the direction of rotation of the vortices. A similar pair of vortices with opposite rotations can be seen in the no vibration case at $t/\tau =0.8$ but they disappear in the case with vibration at a similar time instant. This pair of vortices at the trailing edge indicates the presence of a jet, which favors thrust generation. Figure 15 shows a negative perturbation velocity at $t/\tau = 0.7 \;  \&  \; 0.8$, which implies a motion of the FWMAV model towards the jet due to the self-induced vibration. That is, the whole body is moving towards the counter-rotating vortices, which ebbs the jet effect and consequentially decreases the thrust in the oscillatory case. Similar physics can be observed at 35% of the wingspan, which is shown in Fig. 16. Also, the normalized velocity comparison at 35% wingspan is shown in Fig. 17.

**Figure 18:**
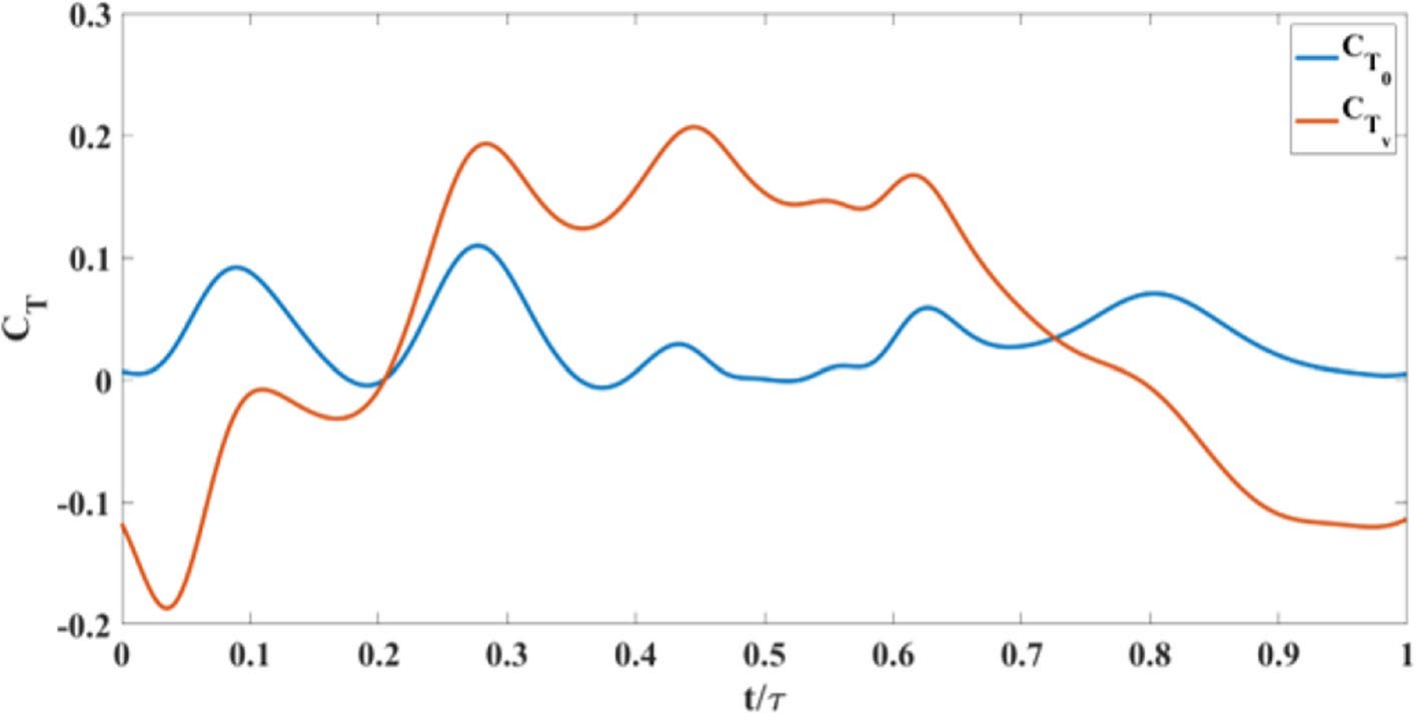
Thrust coefficient comparison for Model A with and without self-induced body vibration.

Figure 18 shows the coefficient of thrust during a flapping cycle for Model A, with and without self-induced vibration. $C_{T_{0}}$ denotes the thrust coefficient based on the aerodynamic model discussed in “Aerodynamic model for 2 wings” section ^41^ without any perturbation, while $C_{T_{v}}$ is the coefficient of thrust when the measured vibration-induced perturbation is applied to the aerodynamic model. Figure 18 shows that approximately in between $t/\tau =0.2 \;  \&  \; 0.7$, $C_{T_{v}}$ is greater than $C_{T_{0}}$. The reason can be seen in the flow visualization. Presented in Fig. 16; that at $t/\tau =0.4 \;  \&  \; 0.5$, in the no vibration case, there is a single trailing-edge vortex (TEV) attached to the trailing edge. By contrast in the oscillatory case, TEV is detached from the trailing edge. The TEVs are shown in green dashed circles. Model A generates thrust using a conventional unsteady lifting mechanism: A TEV is shed whenever there is a change in the wing motion, which changes the wing-bound circulation as well as aerodynamic forces, because of the conservation of circulation. This mechanism has a transient response (Wagner’s effect^42^). Due to this response, the closer the TEV is to the trailing edge, the smaller its strength is compared to the steady value. Figure 16 shows that at $t/\tau =0.4 \;  \&  \; 0.5$, $\Delta V$ is positive, which means that the whole body, due to the perturbation is moving to the left, leaving the TEV detached from the trailing edge. This results in an increase in $C_{T_{v}}$ over a specific duration. But after $t/\tau =0.7$, $\Delta V$ becomes negative and the whole body moves into the jet and loses thrust as discussed previously. This is reflected in Fig. 18 that beyond $t/\tau >0.7$, $C_{T_{v}}$ becomes way less than $C_{T_{0}}$. In the end, the average $C_{T_{v}}$ is less than the average $C_{T_{0}}$ over the flapping cycle.

### Effects of self-induced vibrations on the four-wings model (Model B)

**Figure 19:**
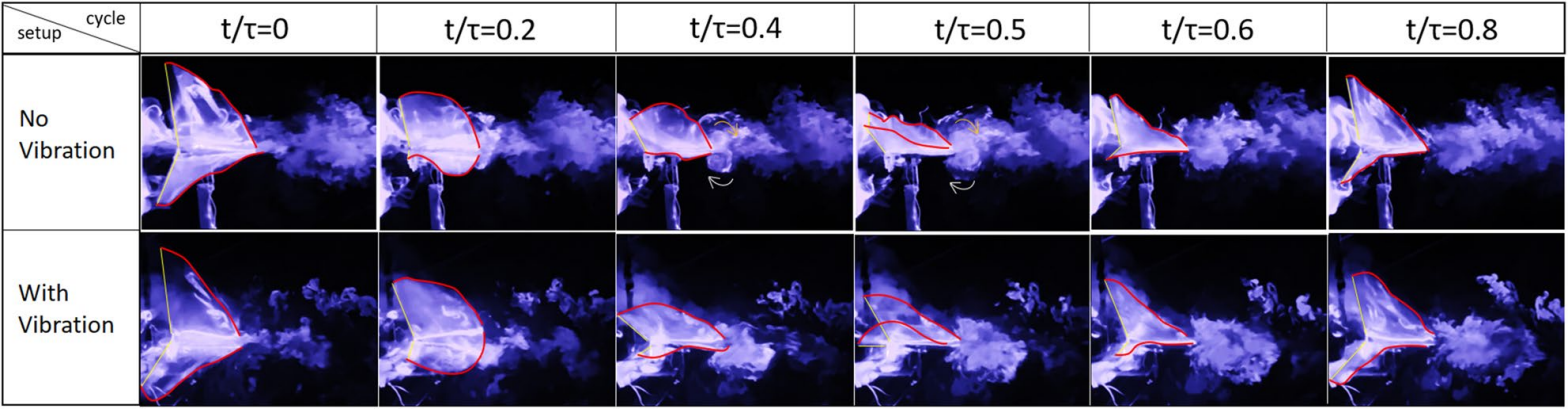
Flow visualization images from oscillatory test (with vibration) and fixed test (no vibration) at 15% spanwise location for Model B.

**Figure 20:**
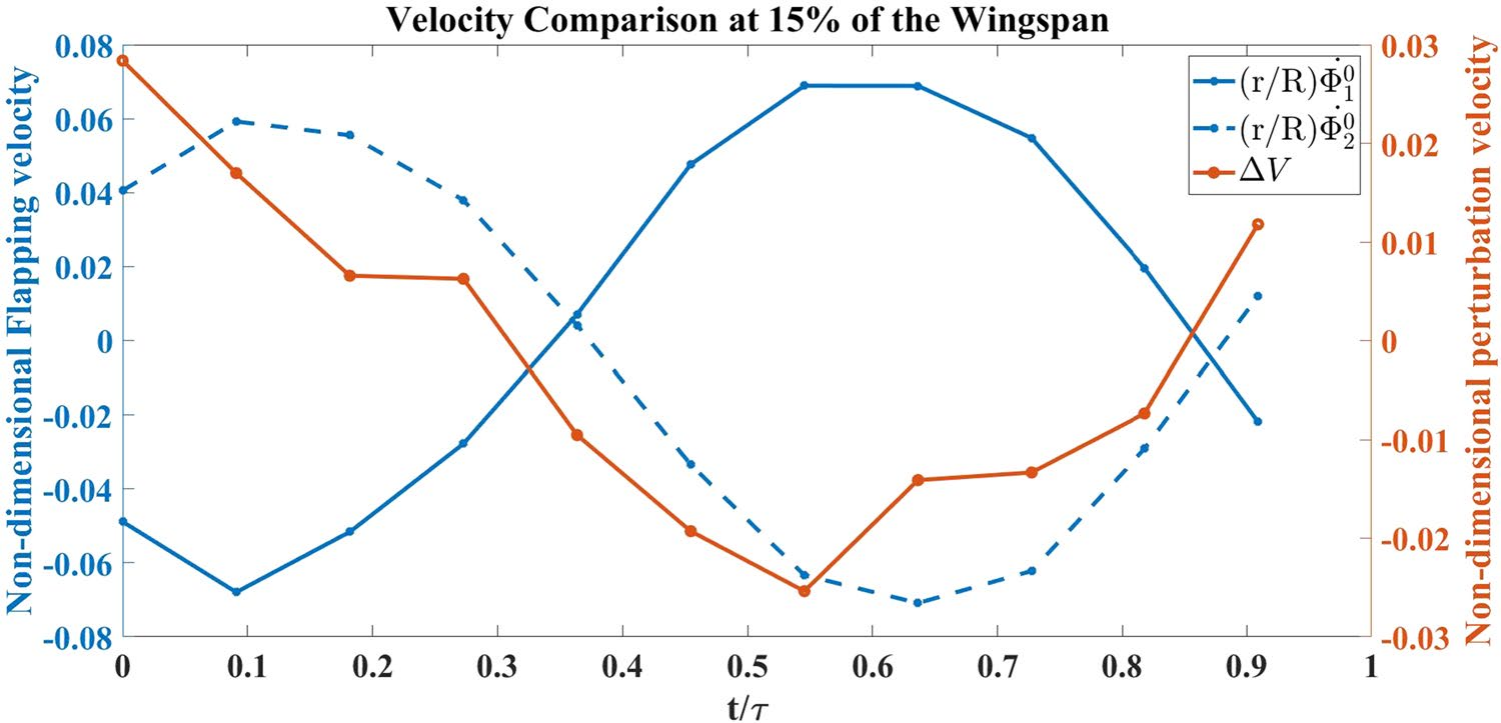
Normalized vibration and flapping velocity at 15% spanwise location for Model B.

**Figure 21:**
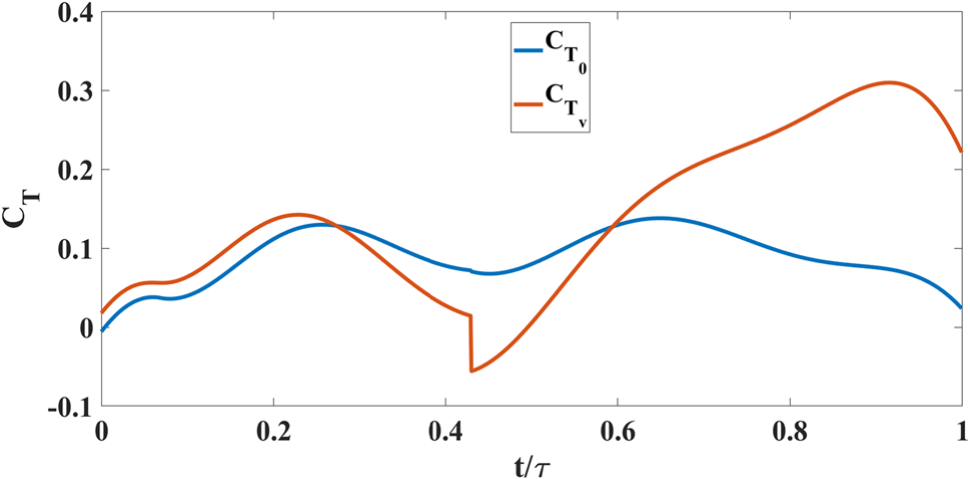
Thrust coefficient comparison for Model B with and without self-induced body vibration.

This sub-section is dedicated to analyzing the effect of self-induced vibration in the flow field of Model B at a 15% spanwise location. As mentioned by Balta et al.^35^, the four-wings mechanism generates thrust through the clap and peel mechanism. During the peel motion, it creates a suction between the wings, which intakes a significant amount of air. During the clap motion, it pushes the air downstream creating a ’jet burst’. Figure 19 shows that for the no vibration case, there are two counter-rotating vortices at $t/\tau =0.4 \hspace{1mm}  \&  \hspace{1mm} 0.5$ which are indicative of the ’jet burst’. However, the figure does not show similar vortices in the oscillatory case at the same instant. Figure 20 shows that the perturbation velocity at $t/\tau =0.4 \hspace{1mm}  \&  \hspace{1mm} 0.5$ is negative. This implies that the FWMAV is moving to the right during this time. This motion of the FWMAV towards the jet decreases the thrust, which can be seen in Fig. 21. In this figure, $C_{T_{v}}$ is the thrust coefficient resulting from the aerodynamic model presented in “Aerodynamic model of 4 wings” section with the perturbation and $C_{T_{0}}$ is the coefficient without the perturbation. We can see that $C_{T_{v}}$ is less than $C_{T_{0}}$ at $t/\tau =0.4 \hspace{1mm}  \&  \hspace{1mm} 0.5$. In contrast, Fig. 20 shows that $\Delta V$ is increasing during the ensuing period which means the flapping robot is moving away from the jet. The jet bursts and the self-induced perturbation takes the vortices away from the trailing edge. Thus it enhances the clapping effect; and the thrust increases significantly, as shown in Fig. 21 after $t/\tau =0.6$, compared to the case with no vibration. This enhanced clapping effect dominates the average $C_{T_{v}}$ over the average $C_{T_{0}}$.

In table 1, the experimental and aerodynamic modeling values of $\frac{\overline{C_{T_{v}}}-\overline{C_{T_{0}}}}{\overline{C_{T_{0}}}}$ are presented in percentage format for flapping frequency 6Hz.

**Table 1:** Percentage of average thrust coefficient change from ideal hovering to the case with perturbation, using aerodynamic modeling and experimental measurement.

	2 wings	4 wings
Experimental	-59.1%	64.4%
Aerodynamic Modeling	-24.4%	55.6%

Finally, it may be prudent to emphasize that the obtained results and conclusions might not be directly extended to biological flyers or FWMAVs in free flight. The constraints in the pendulum setup may limit body motion, which, if allowed, could decrease the resulting aerodynamic loads (particularly for the two-winged model): A flapping wing experiences drag as it sweeps back and forth; this drag is transferred to the body, which causes the body to move opposite to the flapping wing. As such, the speed of the flapping wing with respect to the surrounding quiescent air is less (it is the flapping speed minus the body’s backward speed), which results in smaller aerodynamic loads^22,23^. This interaction is not captured in the pendulum setup because the stroke plane is almost parallel to the pendulum rod; the backward body motion (along the stroke plane) is restrained. From this discussion, it may be concluded that the measured thrust forces in the two-winged model, pendulum-setup case are higher than those in free flight, which does not contradict the general conclusion of the study: body oscillations reduce the averaged thrust in the two-winged model. In the four-winged model, however, the pendulum constraint may not be as restrictive because the instantaneous drag and inertial forces coming from each wing may cancel when transferred to the body (as they move in opposite directions).

## Conclusion

The current study compares two cases of hovering flights. The first case is of an ideal hovering; there is no room for perturbation. The measurement in this case is carried out using the loadcell setup, which is also called the fixed test. The other case of hovering allows for concomitant self-induced vibration due to the oscillatory nature of the thrust force. A pendulum setup, which is also called the oscillatory test, is used to measure the average force in this case. This force measurement includes the effect of the self-induced vibration. These cases are studied using two different flapping wing robots: a two-winged robot or Model A and a four-winged robot or Model B. When these models are tested in the above-mentioned setups, it is observed that the fixed test measures more thrust than the oscillatory test for Model A. The opposite behavior is observed for Model B as shown in Fig. 7. Model B exploits ’clap-and-peel’ for generating thrust, whereas Model A uses the conventional flapping mechanism for the same. Due to the difference in the thrust generation mechanisms, the effect of the perturbation also differs between the two models.

Two well-known aerodynamic models (^33,41^) for thrust generation are used to match the loadcell data by optimizing some unknown parameters. The perturbation velocity, measured using the motion capture system, is applied in the model to study the effect of the induced vibration during the flapping cycle. The perturbation is believed to have some effect on the flow field. To investigate how much impact the vibration has on the flow field, the flow visualization technique is used to look into it. The aerodynamic modeling and the flow visualization are done at the 6*Hz* flapping frequency.

Flow visualization revealed some interesting vortex interactions. In the case with no vibration, Model A enjoys a certain jet effect near its trailing edge. The perturbation wanes its effect by moving the whole flapping robot into the jet. This decreases the overall thrust for Model A in the oscillatory test. For Model B the self-induced vibration enhances the thrust by moving the flapping robot away from the jet. This phenomenon enhances the clapping effect and consequently increases the overall thrust in the oscillatory test. The vortex interactions show how the self-induced vibration has an adverse effect on the thrust generation for a two-winged flapping robot. In contrast, the vibration enhances the effect on a four-winged flapping robot.

### Supplementary Information

Supplementary Information.

## Data Availability

The dataset used and/or analyzed during the current study are available from the corresponding author on reasonable request.
