# Supplementary material for: Thrust enhancement and degradation mechanisms due to self-induced vibrations in bio-inspired flying robots
Source: Sci Rep. 2023 Oct 25;13:18317. doi: 10.1038/s41598-023-45360-4 (PMC10600193; doi:10.1038/s41598-023-45360-4)
Supplement: Supplementary file 1 — Supplementary Information. [file 41598_2023_45360_MOESM1_ESM.pdf]

Appendix of "Thrust Enhancement and Degradation Mechanisms due to Self-Induced Vibrations in Bio-inspired Flying Robots"

Dipan Deb, Kevin Huang, Aakash Verma, Moatasem Fouda, and Haithem E. Taha
University of California Irvine, Irvine, California-92697, USA

I. Friction Measurement

To estimate damping in the pendulum setup, we apply a disturbance in the pendulum angle to the system without the wings. Figure 1 shows the measured response of the pendulum angle due to such a perturbation. From these measurements, we estimate the damped frequency ($\omega_d = 0.744HZ$) and the damping ratio ($\zeta = 0.0127$), using the logarithmic decrement rule.

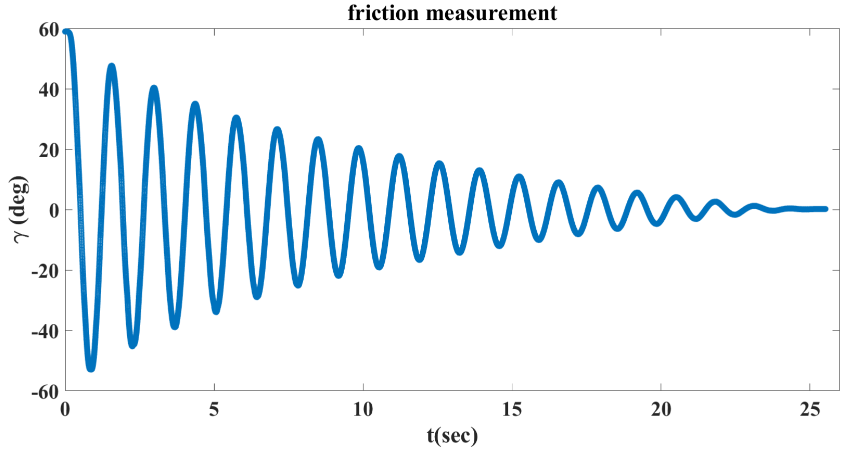

Fig. 1 Free damped oscillation of the FWMAV on the pendulum

II. Structural Response from the loadcell

To obtain a rough estimate of the natural frequency of the structure of the loadcell experimental setup, we perform an experimental run without the wings (so no appreciable aerodynamics). Note that the wings weigh less than 1% compared to the flapping robot. So, removing it is not expected to cause a significant change in the structural frequency of the system (it will definitely change the *aeroelastic* frequency, but this is not our concern here). In this case, zero averaged thrust is expected due to the lack of aerodynamic loads on the system.

Figure 2 shows the time variation of the measured thrust in this case of no wings along with its FFT. The average thrust is close to zero as expected. Moreover, the FFT possesses a peak at 52 Hz irrespective of the value of the “flapping”

frequency.

We use USBPGF-S1 low pass filter from alligator technologies. We choose an eighth-order Butter worth filter with a cut-off frequency of 50Hz. We expect the sharp frequency response of the eighth-order Butter worth filter to attenuate most of the content of the structural response at 52Hz.

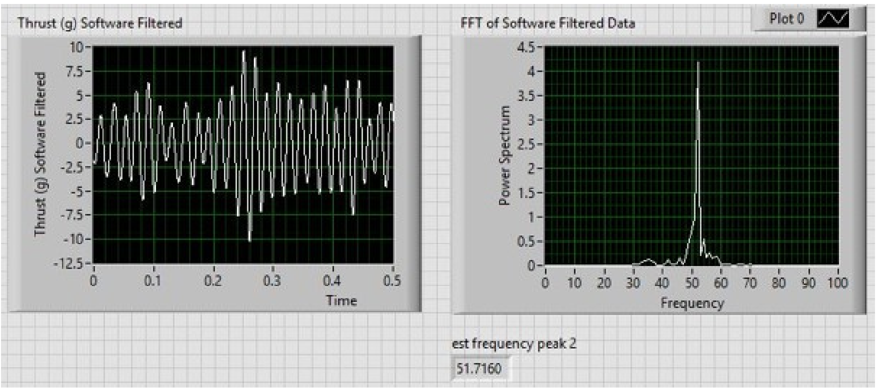

Fig. 2   Structural response from the loadcell
